# Prevalence of chronic respiratory symptoms and associated factors among woodwork workers in Bahir Dar City, Ethiopia; a comparative cross-sectional study

**DOI:** 10.1186/s12890-023-02812-x

**Published:** 2024-01-02

**Authors:** Girmaw Abateneh, Zemichael Gizaw, Mulat Gebrehiwot, Eshetu Abera Worede

**Affiliations:** Department of Environmental and Occupational Health and Safety, Institute of Public Health, University of Gondar, Gondar, Ethiopia

**Keywords:** Respiratory symptoms, Wood dust, Woodwork workers, Work-related exposure

## Abstract

**Background:**

Exposure to wood dust can cause respiratory symptoms, like cough, phlegm, breathlessness, and chest pain, reduce lung function.

**Objective:**

The objective of the study was to assess the prevalence of respiratory symptoms and associated factors among woodwork workers in Bahir-Dar city, Ethiopia.

**Methods:**

A comparative cross-sectional study was employed among 229 exposed and 228 unexposed groups. Participants for the study were selected using simple random sampling technique. The chronic respiratory symptoms were assessed using a questionnaire adopted from the American Thoracic Society. The data was entered using Epi-Data version 4.6 and export to SPSS version 22 for analysis. Poisson regression, Multivariate linear regression and multivariable logistic regression analysis were used to identify factors associated with woodworkers, general population and in pooled analysis.

**Result:**

The prevalence of having at least one chronic respiratory symptom was higher among exposed group (59.4%) than unexposed group (18.0%) with PR = 3.03(95%CI: 2.45, 4.45). In woodworker; Not taking health and safety training(5.15,95%(CI:1.93–13.76),primary educational(3.85,95%,CI:(1.1,13.47), not using Mask(6.38, 95%CI:(2.69–15.76) & number of families(3.05,95%,CI:1.04–9.028), In general population; Number of family members(2.75, 95%CI:1.1–7.19)& lower monthly income (3.3, 95%CI: (1.49–7.4), and In pooled analysis; wood dust exposure status 14.36 95%, CI:(7.6–27.00),primary education(2.93,95%CI:1.24–6.92), number of families(3.46,95%CI:1.8–6.64), lower monthly income(2.13,95%CI:1.19–3.81), & smoking (6.65, 95%CI:1.19–36.9) were associated with respiratory symptom.

**Conclusion:**

Prevalence of respiratory symptoms was higher among exposed group than unexposed group. Reduced wood dust exposure status, Provision of occupational safety and health training, use of respiratory protective devices is recommended to reduce respiratory symptoms among woodwork workers.

## Background

Wood dust and its components are known to cause respiratory symptoms and have sensitizing and irritating properties to mucous membranes [1]. Work-related respiratory illnesses are the major global public health problems, accounting for up to 30% of all work-related diseases and up to 50% among workers in high dust-generating industries or work activities [2]. Inhaling wood dust and its various components cause respiratory disorders and irritates the mucous membrane of the airways, which are exhibited by a variety of signs and symptoms in exposed workers [3]. Workers in the woodwork industry are at high risk of being exposed to wood dust, and the development of various respiratory symptoms [4].

The European Union and Occupational Safety and Health Administration(OSHA) regulations stipulate a marginal value for wood dust exposure level [5], and such regulation is single 8-hour time weighted average(TWA) of 5 mg/m^3^ and a short term exposure limit(STEL) of 10 mg/m^3^ for all hard wood and soft wood dusts [6]. Occupational exposure to wood dust has been linked to a variety of respiratory disorders, including asthma, allergic rhinitis, chronic bronchitis, and lung function impairment [4, 7].

Two million woodworkers around the world are exposed to wood dust every day, and increase prevalence of respiratory tract diseases [8]. Wood dust is produced during the process of the wood furniture making which involves sanding, sawing and drilling either manually or with machines [9],and globally the prevalence of respiratory disease in such occupation is reported in the range of 5.6–18%, and in Africa is between 3 and 7% [10]. Some studies showed a high prevalence of respiratory symptoms like cough, phlegm, chest tightness, wheezing, and breathlessness among exposed group relative to unexposed groups [11–13]. Similar studies also reported a high prevalence of respiratory symptoms among wood dust exposed group [7, 14–16].

Respiratory symptoms are associated with different factors such as socio-demographic variables like gender [17–19], Socio-economic status, level of education, types of occupation [20–22], family size [23], family history of respiratory illness [19, 24, 25], behavioral factors like smoking [19, 26, 27], working condition factors like duration of exposure [28], and working department [17], utilization of personal protective equipment such as dust mask [22], and effective housekeeping and exhaust ventilation [14, 29], health and safety training [21]. But there is limited evidence on the prevalence of respiratory symptoms and associated factors among woodwork workers in Ethiopia, especially in the Bahir Dar City. Therefore, this study aimed to assess the prevalence of work-related respiratory symptoms and associated factors among woodworkers in Bahir-Dar city, Ethiopia.

## Methods and materials

### Study design and period

A comparative cross-sectional study was conducted in Bahir Dar city among woodwork workers from March to April 2021.

### Study area

The study was conducted in Bihar Dar city, which is the capital city of Amhara regional State, and it is found 565 km from Addis Ababa, the capital city of Ethiopia. Based on the Central Statistical Agency of Ethiopia’s (CSA) 2007 Census, the Bahir Dar Special Zone had a total population of 221,991, of which 108,456 were male and 113,535 were female; 180,174 or 81.16% are urban residents, the rest living in rural kebeles around Bahir Dar [30]. In the city, there are 280 woodwork industries with a total of 1600 workers (in average there were 6–10 workers in one woodwork industry. The common types of wood that used for furniture making were Wanza (local language) tid(timber), and medium density fiber board(mdf).

**Fig. 1:**
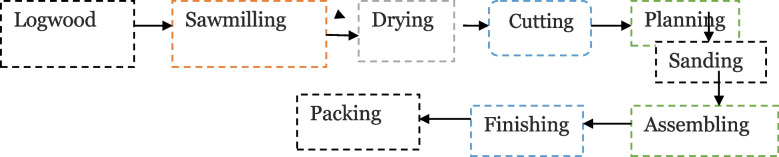
Work process flowchart of woodwork industries in Bahir Dar city, 2021

The woodwork production process begins with logwood, from which various raw materials are stoked, and it is distributed to the sawmill, which splits the wood to the required size. After splitting the material to the required size, the moisture content is lost through drying to produce a good product. The wood will then be cut and split using a cutter saw or a circular saw depending on the size product required. Then, sanding is performed to obtain components that have been refined with the same size and smoothness prior to the assembling process, which involves joining different components together to produce finished goods. The final process in the woodworking industry is finishing, which is layering wood surface with the purpose of making it beautiful, and products move to packaging area to add some accessories like key, handle, real, soon (Fig. 1).

### Source and study population

This study includes all woodwork workers (exposed group) who had worked 1 year and above in the woodwork industries, and the general population (unexposed group) who live nearby with those factories around 2.5-km from the woodwork industries and had never worked in the woodwork industry [31].

### Inclusion criteria

In this study, participants with 1 year and above work experience were included in the study.

### Exclusion criteria

Both woodwork workers (exposed) and the general population (unexposed groups) with a history of pulmonary disease, abdominal tumor, heart failure, or recent surgery or thorax procedures were excluded from this study. Unexposed groups were exclude that had wood dust exposure history.

### Sample size determination

The sample size was done for both prevalence and associated factors. For the prevalence, sample size was done using the double population proportion formula, by considering the proportion of respiratory symptoms for the exposed(44.2%), and unexposed(14.3%) groups in the manufacturing industry [3], 95%CI, and 80% power respectively.

***n*** **1** ***= n*** **2** ***=*** (**Z*α***/**2** ***+*** **Z*β***)^**2**^ ***×*** [***p***(**1** ***− p*1**) ***+*** (***p*2**(**1** ***− p*2**)]

$${\left(P1-p2\right)}^2$$

$$n={\left(1.96+0.84\right)}^{2\Big[}\left(0.442\ \left(1-0.442\right)\right.+\left(0.143\left(1-0.143\right)\right.\Big]=32$$

$${\left(0.442-0.143\right)}^2$$

The sample size was 32 exposed and 32 non-exposed groups and after adding a 10% non-response rate, the total sample size was 70 for both populations [3]. The sample size for associated factors was done using Open Epi info software version 7,by considering 95% confidence interval, and 80% power, ratio exposed to unexposed group is 1:1, and factors from the previous study; smoking and proportion among exposed (7.3%) and unexposed group (3.8%) [2] and the sample size was 348 for both groups. The other factor was occupational safety and health training with the proportion of exposed (8%) and unexposed(3.8%) with 10% non –response rate, the total sample size was 472 (236 for exposed and 236 unexposed groups [2]. Therefore, the sample size for factors is higher than a sample size of the prevalence and the final sample size for this study was 472.

### Sampling procedure

In Bahir Dar City, there are nine sub-cities, and from the nine sub-cities, by using lottery method three sub cities such as Dagimawie Minilk, Fasilo, and Tewodrose sub-cities, with a total of 107 woodwork factories were selected. Samples in the exposed and unexposed groups were proportional allocated and simple random sampling technique was used to select study participants. The study population from the general community was recruited from the selected sub-cities found around 2.5 k meters from woodwork factories (assuming less wood dust exposure).

### Data collection procedure

The questionnaire was adapted and modified from a validated standardized questionnaire from the American thoracic society(ATS) to assess respiratory symptoms in adult populations [32]. The questionnaire had the following components: Socio-demographic characteristics, respiratory symptoms, family history of respiratory diseases, behavioral factors including housing condition with types of energy used for cooking, and working conditions factors. The data was collected through face-to-face interviews. Observational checklist also was used to see safe work practices like respiratory protective device utilization and workplace ventilation.

### Data quality assurance

The quality of data was assured through careful design, languge translation, and retranslation of the questionnaire. Two-days training were given for data collectors and supervisors. A pre-test was carried out before the actual data collection in Geshi- Abay sub city to check the competency of the data collectors, and the reliability and validity of the data collection tools and necessary corrections were taken accordingly. Data were collected by five public health professionals.

### Study variable

#### Dependent variables

Chronic respiratory symptoms.

#### Independent variables

There are different factors that affect respiratory symptom including socio-demographic variables (age, sex, education, marital status, income level), previous and family history of respiratory disease, behavioral factors (smoking habit, PPE, types of energy source at home), work-related factors including working condition factors such as wood dust exposure status, and OSH training, work experience, work place ventilation, periodic medical check-up & pre-medication.

### Operational definition

#### Chronic respiratory symptoms

Workers with one or more symptoms of cough, phlegm, wheezing, chest tightness, and breathlessness last at least 3 months in 1 year [3].

#### Past chronic respiratory disease

One or more respiratory diseases like chronic bronchitis, emphysema, tuberculosis (TB), heart disease, chronic sinus, asthma, and lung cancer that could be developed before the current working position and identified by physicians [19].

#### Cough

In this study, Cough is defined as it occurs 4 to 6 times a day for most days of the week (≥ 4 days) for at least 3 months a year [21].

#### Phlegm

Participants were considered to have chronic Phlegm if they answered yes/ to at least one of the following four questions; Phlegm first thing in the morning, Phlegm during the day or night, phlegm as much as four to six times a day in a week or phlegm for most days as much as three consecutive months during the year [14].

#### Breathlessness

Participants were considered to have chronic breathlessness if he/she was troubled by shortness of breath when hurrying on level ground or walking up a slight hill, or getting shortness of breath when walking at /her own pace on level ground [33].

#### Chest pain

Chest discomfort that has held of phlegm workers’ jobs in the past 1 year or above [17].

#### Wheeze

Whistling breathing during the respiratory cycle perceived by the respondents at least 3 months in a year [3].

#### Current smokers

Workers who smoked at the time of the study or someone who smokes cigarettes every day or every few days) [34].

#### Ever smoker

A person who smoked at least 100 cigarettes in his entire life [19].

#### Wood dust

Wood dust is an accumulation of any wood particulate that is generated during the processing or handling of wood. When this dust becomes airborne it may be inhaled by workers [35, 36].

#### Data management and analysis procedure

The data were cleaned and checked using frequency table and entered into the Epi-Data version 4.6,EpiData Association, Denmark, Europe [37] and exported to SPSS version 22 for further analysis. Descriptive statistics, Poisson regression models with robust variance were employed to estimate the prevalence ratios (PR) and corresponding 95% confidence intervals (CI) [38]. Logistic regression analysis was used to identify whether exposure variables are significantly associated with outcome variables or not. Thus, variables in the bi-variable analysis with *p* ≤ 0.2 were included in the multivariable analysis by adjusting confounding variables. We considered variables as significant independent factors based on Adjusted Odds Ratio (AOR) with 95% CI and *P*-value of < 0.05. The *P*-value of < 0.05 was considered as statistically significant.

## Results

### Socio-demographic characteristics of the study participants

A total of 457(229 exposed and 228 unexposed groups) respondents with a response rate of 96.8% participated. The mean ± (SD) age of the participant among exposed and unexposed groups was 33 ± 6 and 35 ± 7 years respectively. The majority of the study participants were males. Less than half (43.2%) of the exposed group have secondary level education, and more than a quarter (31.1%) of the unexposed group attend secondary educational level (Table 1).

**Table 1:** Socio-Demographic characteristics of respondents in the woodwork workers in Bahir Dar city, Northwest, Ethiopia, 2021(*n* = 457)

Variable	Response	Exposed *n* = 229	Unexposed *n* = 228	*P* value
Sex	Male Female	210(91.7) 19(8.3)	189(82.9) 39(17.1)	0.001
Age	< 29 ≥29	63(27.5) 166(72.5)	56(24.6) 172(75.4)	0.47
Level of education	Primary education Secondary school Diploma &above	64(34.9) 108(43.2) 57(21.8)	134(58.8) 71(31.1) 23(10.1)	0.001
Income per month	≤4300(ETB) > 4300(ETB)	97(42.4) 132(57.6)	146(64) 82(36)	0.001
Marital status	Married Single	164(71.6) 65(28.4)	197(86.4) 31(13.6)	0.001
No of family	≤ 3 > 3	139(60.7) 90(39.3)	103(45.2) 125(55.8)	0.001

### Behavioral characteristics of study participants

In this study, nearly two third(65.5%) of exposed groups and the majority(83.8%) of the unexposed groups used electricity as the source of energy at home, and 8.3% of exposed groups and 1.3% of unexposed groups ever cigarette smokers (Table 2).

**Table 2:** Behavioral characteristics of study participants in Bahir Dar city Northwest, Ethiopia, 2021

Variable	Categories	Response	Unexposed n (%)	*P* value
		Exposed n (%)		
Types of energy used	Electric Charcoal Fire kerosene, others	150(65.5) 73(31.8) 4(0.4) 3(1.4) 2(0.9)	191(83.8) 35(15.3) 2(0.9) ----- -----	0.56
Now smoker	Yes No	11(4.8) 218(95.2)	1(0.4) 227(99.6)	0.001
ever smoker	Yes No	19(8.3) 210(91.7)	3(1.3) 225(98.7)	0.001
Ever worked in other dust types of work	Yes No	5(2.2) 224(97.8)	8(3.5) 220(96.5)	0.24

#### Work-related characteristics of study participants

More than half (58.5%) of woodwork workers had 5 and below years of work experience. The average (±SD) work experience of woodworkers in woodwork industry was 5.62 ± 3.37 years. The majority (84.7%) of workers had worked 6–8 hours per day. One-fourth 58(25.3%) of participants had taken occupational health and safety training. Almost 40 % (39.7%) of participant had utilized personal protective equipment and the rest were not used personal protective equipment.

#### Prevalence of chronic respiratory symptoms

After Adjusting, sex, age, educational status, monthly income, work experience, working hours per day, dust exposure in previous employments, biofuel energy use, use of PPE, and OSH training, and the prevalence of at least one chronic respiratory symptom was higher among exposed group (59.4%) than unexposed group (18.0%)(PR = 3.03(95%CI: 2.45, 4.45). The prevalence ratio of cough (PR = 2.37(1.56, 3.6), phlegm (PR = 2.39(1.46, 3.89), wheezing (PR = 7.3(3.17, 16.8), Shortness of breath (PR = 4.5(3.01, 6.86), and Chest pin (PR = 4.97(2.45, 4.45). The prevalence of all the respiratory symptoms was high significantly for the exposed group than non-exposed groups (*p*-value < 0.05) (Table 3).

**Table 3:** Prevalence of respiratory symptoms among exposed and unexposed group in Bahir Dar city, northwest Ethiopia, 2021

Types of respiratory symptom	Exposed group *n* = 229	Unexposed group *n* = 228	PR 95% CI	p value
Cough	62(27.1)	26(11.4)	2.37(1.56, 3.6)	0.001
phlegm	48(21)	20(8.8)	2.39(1.46,3.89)	0.001
Wheezing	44(19.2)	6(2.6)	7.3(3.17, 16.8)	0.001
Shortness of breath	105(45.9)	23(10.1)	4.5(3.01, 6.86)	0.001
Chest pin	55(24)	11(4.8)	4.97(2.67, 9.3)	0.01
At least one respiratory symptom	136(59.4)	41(18)	3.3(2.45, 4.45)	0.001

*PR* Prevalence of Ratio, *I* Confidence Interval

#### Factors association with respiratory symptoms among woodwork workers (model I)

Factors such as sex, income, age, marital status, and educational level, and family size, use of PPE, cigarette smoking, work experience, occupational health, and safety training were found candidate (*p*-value ≤0.2) for multivariable logistic regression model. But, variables such as use of respiratory protective equipment (RPE), educational status, family size, and, occupational health and safety training were found associated with respiratory symptoms among woodwork workers in multivariable logistic regression model.

The respiratory health symptoms was 5.15 times higher among woodwork workers who did not take occupational safety and health training when compared to workers who did take occupational safety and health training (AOR = 5.15, 95%CI: 1.93–13.76).The respiratory health symptoms among woodworkers who did not use respiratory protective equipment was 1.79 times higher than workers who used respiratory protective equipment (AOR = 6.38, 95%(CI: 2.69–15.76). The respiratory health symptom among participants with lower education was 3.85 times higher when compared to those participants with diploma and above education (AOR = 3.85(95%CI: 1.10–13.47). The respiratory symptoms among woodworkers who had more than 3 family members was 3.06 times higher than workers who had less than or equal to 3 family members (AOR = 3.06, 95%CI: 1.04–9.02) Table 4).

**Table 4:** Factors associated with respiratory symptoms in exposed group in Bahir Dar city northwest, Ethiopia, 2021(*n* = 229)

Variable		Respiratory symptoms		COR(95%CI)	AOR(95% CI)	*P* value
		Yes	No			
Sex	Male Female	131 5	79 14	4.6(1.61–13.38) 1	1.3 (0.31–5.09) 1	
Age(year)	< 29 ≥29	20 116	43 50	1 4.98(2.7–9.32)	1 0.88(0.32–2.94)	
Marital status	Married Single	117 19	47 46	6.63(3.20–11.35 1	0.76(0.26–2.22) 1	
Educational level	Primary Secondary diploma& above	67 58 11	13 41 39	18.2(7.47–44.7) 5.0(2.3–10.9) 1	3.85(1.10–13.47) 0.41(0.187–1.99) 1	< 0.05
Monthly Income	≤4300 > 4300	36 100	61 32	0.19(0.11–0.34) 1	2.28(0.75–6.9) 1	
Family size	≤ 3 > 3	57 79	82 11	1 10.33(5.1–21.13)	1 3.06(1.04–9.02)	< 0.05
Ever smoker	Yes No	14 122	5 88	2.02(0.702–5.81) 1	0.46(0.11–1.8) 1	0.09
Use of PPE	Yes No	27 109	64 29	1 8.90(4.85–16.37)	1 6.38(2.69–15.76)	< 0.001
Work experience	≤5 Year > 5 years	52 84	82 11	1 12.04(5.87–24.69)	1 2.45(0.96–6.45)	
OSH training	Yes No	12 124	46 47	1 10.11(4.93–20.75)	1 5.15(1.93–13.76)	< 0.001

*AOR* Adjusted Odds Ratio, *COR *Crude Odds Ratio, 1.00 = Reference, Osmer and Lemeshow test = (*p* 0.25)

#### Factors association with respiratory symptoms among unexposed group (model II)

Risk factors like sex, family size, educational status, and monthly income, were candidate (p value≤0.2) for multivariable logistic regression. But, variables such as monthly income and family size were associated with respiratory symptoms among the general population. The respiratory health symptom was 2.75 times higher among participants who had more than three family members when compared to participants who had less than three family members (AOR = 2.75, 95%, CI: (1.1–7.19).The respiratory health symptom was 3.3 times higher in participants who had lower monthly income when compared to participants who had higher monthly income (AOR = 3.3, 95%CI: (1.49–7.4) (Table 5).

**Table 5:** Multivariable analysis and factors association with respiratory symptoms of the unexposed group (*n* = 228)

Variable		Respiratory symptoms		COR(95%CI)	AOR(95% CI)	*P* value
		Yes	No			
Sex	Male Female	40 1	149 38	10.2(1.4–76.5) 1	6.26(0.79–49.33) 1	
Family No	≤ 3 > 3	7 34	96 91	1 5.12(2.16–12.14)	1 2.75(1.1–7.19)	< 0.05
Educational level	Primary Secondary Diploma &above	28 10 3	106 61 20	1.76(0.49–6.35) 1.1(0.27–4.37) 1	1.47(0.35–6.21) 0.93(0.21–4.18) 1	
Income	≤4300 > 4300	13 28	133 54	5.31(2.55–11.0) 1	3.3(1.49–7.4) 1	< 0.05

1, Reference group and, *AOR* Adjusted Odds Ratio, *COR *Crude Odds Ratio

#### Factors association with exposed group and unexposed group (model III)

In a pooled model, sex, age, wood dust exposure status, family size, marital status, educational level, income, smoking status were candidate (*p* value≤0.2) for multivariable logistic regression analysis. But, variables such as wood dust exposure status, family size, income, and educational level and Ever smoker were significantly associated with the respiratory symptoms in poled analysis.

The respiratory health symptom among exposed group was 14.36 times higher among exposed group when compared to unexposed groups (AOR = 14.36 95%CI: (7.6–27.0).The respiratory health symptom was 3.46 times higher among participants who had more than three family members when compared to participants who had less than three family members (AOR = 3.46, 95%, CI: (1.8–6.64). The respiratory health symptom among participants who had family size more than three was 3.46 times that of participants with less than three family sizes(AOR = 3.46, 95%CI:(1.8–6.64)). The respiratory health symptom among participants with lower education was 2.93 times higher when compared to those participants with diploma and above education (AOR = 2.93(95%CI: 1.24–6.92). The respiratory health symptom among ever smokers was 6.65 times higher when compared to non-smokers (AOR = 6.65, 95%CI: (1.19–36.9) (Table 6).

**Table 6:** Multivariable analysis for factors associated with respiratory symptoms among the exposed group and unexposed group in Bahir Dar city, Northwest, Ethiopia, 2021

Variable		Respiratory symptoms		COR(95%CI)	AOR(95% CI)	*P* value
		Yes	No			
Exposure status	Exposed unexposed	136 41	93 187	6.67(4.34–10.24) 1	14.36(7.6–27.0) 1	0.001
Sex	Male Female	171 6	228 52	6.5(2.73–15.48) 1	2.44(0.92–6.47) 1	
Age	< 29 > 29	20 157	99 181	1 4.29(2.54–7.27)	1 1.06(0.51–2.44)	
Family number	≤ 3 > 3	64 113	178 102	1 3.08(2.08–4.55)	1.00 3.46(1.8–6.64)	0.001
Marital status	Married Single	158 19	203 77	3.15(1.83–5.43) 1	1.7(0.75–3.96) 1	
Educational level	Primary Secondary Diploma &above	95 68 14	119 102 59	3.36(1.77–6.39) 2.81(1.45–5.43) 1	2.93(1.24–6.92) 1.5(0.56–3.00) 1	< 0.05
Income	≤4300 > 4300	49 128	194 86	0.17(0.12–0.26) 1	2.13(1,19–3.81) 1	< 0.05
ever smoker	Yes No	17 160	5 275	5.84(2.15–16.14) 1	6.65(1.19–36.9) 1	< 0.05
Now smoker	Yes No	9 178	3 277	4.95(1.32–18.53) 1	0.29(0.03–2.69) 1	

*AOR* Adjusted Odds Ratio, *COR *Crude Odds Ratio, 1.- Reference, hosemer and lemshaw goodness of fit, 0.687

## Discussion

In this study, the overall prevalence of respiratory symptom was higher among the exposed group (59.4%) than unexposed groups (18.0%).This finding is similar to other studies conducted in Europe, Macedonia, [11], Iran [12], Thailand [14], Nigeria [16], and Ethiopia [2, 7]. All of these studies reported that the prevalence of respiratory symptom was higher among the exposed group than in the unexposed groups. This might be due to exposure to dust from woodwork being higher among exposed group than in unexposed groups. Hence, occupational exposure to wood dust has been linked with respiratory symptoms among woodworkers, and the presence of higher prevalence of respiratory symptoms compared with the unexposed groups. On the other hand, this study found higher prevalence of chest pain (13.5% vs 10.8%), phlegm (15.2% vs5.4%), wheezing (8%), and breathlessness (10.5%vs 8.1%) than a study conducted in Europe among woodwork workers [21]. The possible reasons might be due to difference in the implementation of effective dust control measures like natural and artificial exhaust ventilation and utilization of personal protective equipment (PPE) such as dust mask. But the findings of this study was lower than a study conducted in India [39]. The difference might be due to work experience (duration of exposure), utilization of personal protective equipment, and difference in the wood dust exposure level of woodwork workers between the two countries.

In this study associated factor like worker’s exposure status (exposed and unexposed), family size, income, OSH training, use of PPE, and smoking status were significantly associated with respiratory symptoms. In this study the respiratory symptom was more likely among exposed group when compared to unexposed group, and this finding is in agreement with studies conducted in Iran among woodwork workers [40], and Indian sawmill workers [39], and the respiratory symptoms were significantly more prevalent among exposed groups than unexposed group [40].

In this study, the respiratory symptoms were higher among study participants having more than three family members when compared to participants who had less than or equal to 3 family members. This finding is in line with a study conducted in New Zealand [23], and the possible justification might be due to different characteristics of family members, insufficient ventilation, increasing exposure to the infectious agent during social life, overcrowding and difference in lifestyles. Moreover, other factors related to respiratory symptoms was cigarette smoking and were higher among ever smokers when compared to non-smokers and this finding is consistent with studies conducted in Spain [41], South Carolina, US [26], and Iran [42].

In this study, the other significant variable was occupational safety and health-related training, and the probability of having respiratory symptoms was higher among study participants who did not take occupational health and safety training when compared to workers who did take occupational health and safety training. This finding is consistent with other studies conducted in Egypt, and Ethiopia [2, 21, 43]. The possible justification is that training about occupational safety and health improves workers’ attitudes towards respiratory health issues and provides skills and knowledge to implement prevention strategies for occupational hazards (wood dust) in their workplaces.

In this study the respiratory symptoms was more likely among woodworker who did not use respiratory protective equipment (RPE) when compared to workers who used respiratory protective equipment(RPE). This finding is similar to studies carried out in Thailand and Ethiopia [14, 22]. On the other hand, this finding contradicts the finding from the Dejen cement factory, in Ethiopia [21]. This difference could be due to the differences in the amount and kind of dust produced and the quality of respiratory protective equipment provided.

This study also found that the respiratory symptoms of woodworkers were linked with a lower educational level, and this finding was supported by a study performed in Ethiopia, [21] which described that respiratory symptoms were associated with a lower educational level. This might be because higher education provides individuals with skills and knowledge about how to safeguard their health from work-related exposure to health hazards [21].

Moreover, in this study participants with better household income were fewer to have respiratory symptoms when compared to workers with low house hold income. The possible justification might be due to participants with better household income have better living standards and better quality of life, and reduced respiratory symptoms.

### Limitation of study

One of the limitations of this study is the healthy workers effect; workers who developed the symptoms may have quit the job. The use of the questionnaire method was also another limitation of this study because it may cause participants to recall bias and interviewer bias.

## Conclusion

The prevalence of respiratory symptom was higher among exposed group when compared to the unexposed group. In pooled analysis, wood dust exposure status, having more than three family number, and cigarette smoking in the past were associated factors for respiratory symptom. Good work practice to reduce wood dust generation at the source is needed, OSH training, modifying behavioral factors and use of respiratory protective devices are recommended to reduce wood dust exposure and respiratory symptoms among woodwork workers.

## Data Availability

The datasets used and/or analyzed during the current study are available from the corresponding author upon reasonable request.
